# A pilot study on the pattern of COVID-19 information sources and its associated factors among the public in Qatar: a cross-sectional survey

**DOI:** 10.5339/qmj.2025.47

**Published:** 2025-07-05

**Authors:** Wafa Mohammed Ahmed, Ayman Aldahshan, Mohamed Abdien, Iheb buogmiza

**Affiliations:** 1 Department of Community Medicine, Hamad Medical Corporation, Doha, Qatar; 2 Department of Preventive Medicine, Hamad Medical Corporation, Doha, Qatar; 3 Family and Community ‘Medicine Residency Program Primary Health Care Corporation; 4 College of Medicine, QU Health, Qatar University, Doha, Qatar; 5 College of Medicine, Sousse University, Sousse, Tunisia *Email: WAhmed8@hamad.qa

**Keywords:** COVID-19, information sources, seeking behavior, control measures dissemination, public preferences

## Abstract

**Background:**

During the coronavirus pandemic, many people looked for information on COVID-19 through social, official, and traditional media sources. This caustic situation resulted in panic among the public, leading to many false news, conspiracy theories, and magical cures being spread among the public at an alarming rate. Controversial theories about the validity of vaccination and non-adoption of disease control resulted in a slow disease control rate and adverse impacts on global health and the economy. Recent studies have shown that health information could safely guard mental health during the COVID-19 pandemic. To adopt a disease control strategy, it is essential to identify the public confidence in information sources and the most efficient media to disseminate intervention messages to the public. The main objectives of this study were to identify from 12 alternative information sources the most utilized COVID-19 source of information, the most trusted information source, and the factors associated with high trust in a given source.

**Methods:**

A cross-sectional online survey with 442 participants aged 18 years and above was conducted to assess public views and preferences on sources of information for COVID-19. Data was analyzed using descriptive statistics, correlation, and logistic regression analyses. Bloom’s scale, composite score preference, and chi-squared test were determined for result evaluation purposes.

**Results and Conclusion:**

The study findings suggest that the Ministry of Public Health is a widely trusted information source that offers a potentially effective tool for shaping, designing, and disseminating information messages to shape public attitudes toward control of COVID-19 and enhancement of preventive actions. By offering insight into the pattern of COVID-19 information sources and its associated factors among the public in Qatar, the study formulated a set of recommendations for health decision-makers to set packages to help manage and control the spread of coronavirus.

## BACKGROUND

The pandemic coronavirus disease 2019 (COVID-19) affected global health and the economy almost more than 4 years ago.^1^ However, its impacts did not stop and are accelerated by an ongoing upsurge from new strains of the COVID-19 virus. To control such disease, it is thus essential to develop a control protocol consisting of both preventive and curing measures. The preventive measures require the adoption of the most effective source of information delivery and dissemination to the general public. Recent studies have demonstrated that reliable health information can effectively protect mental health during the COVID-19 pandemic.^2^ During pandemics, the public is at high risk of facing a circulation flow of contradicting information. This requires evaluating the reliability of information sources, the public’s preferences, how to disseminate infection control messages, and how to predict and utilize the most effective source of information.

In response to the domination of many unknowns during the invasion of a health pandemic, the demand for information increases, and consequently, the public resolves to the sources they mostly trust.^3^ This ranges from news websites to academic, scientific, and sophisticated sources (scholarly articles on healthcare workers), including official sources (Ministry of Health), media sources (television (TV), and newspapers, including news websites). However, the dilemma is how to select the most effective information source amenable to formulate and dissipate disease control messages to shape people’s attitudes. Given the wide range of available communication channels, evidence shows that people’s utilization of these sources tends to change over time.^4^ Historically, traditional news media have been the most preferred source of information during a health crisis,^5^ while a recent study showed that social media platforms or online news sites were the most predominant sources.^6^ The World Health Organization (WHO) declared that the COVID-19 pandemic has been accompanied by a so-called “infodemic” of misinformation, i.e., too much information including false or misleading information that makes reliable and trustworthy sources difficult.^7^

It is thus essential to define the information sources working at present in the community and direct them to build trust in the health authority protocol of evidence-based measures. In particular, traditional media (such as television and newspapers) and social media were reported to play an important influencing role in communicating evidence-based information to the public.^3,8^

The hypothesis of the high capability of a given source of information to communicate and engage the community through traditional channels, such as television, radio, and text messages, as well as technology and digital health programs, needs to be tested.^9–11^

During pandemics, different demographics face unique challenges in accessing and interpreting health information, which might result in health disparities due to factors such as age, socioeconomic status, education, language barriers, and digital access.

The main objectives of this study were to (1) identify public preferences and trust for information sources on COVID-19, which may help health authorities plan successful preventive and disease control intervention strategies; (2) identify the factors associated with high trust in different sources of information; and (3) identify the socioeconomic and demographic factors associated with each information sources to aid in obtaining a deeper understanding of preferred information sources to enable the public health officials for extending efforts to reach a broader audience.

## METHODS

This study was based on an extensive search of literature bearing in mind the health belief model’s core constructs, mainly perceived disease susceptibility and trust, and its role in the adoption of preventive behavior.

The questionnaire consisted of twenty questions arranged in five primary sections, developed by the study investigators following an extensive literature review. The first section is devoted to personnel information on respondents’ seven socioeconomic and demographic characteristics (Q1–Q7): age., sex, education levels, occupation, marriage status, nationality, and income level. The second section assessed the factors influencing the probability of disease susceptibility, housing condition, history of disease risk factors, and vaccination status (Q8–Q15). The third section assessed sources of information on COVID-19 using 12 multiple-choice questions prepared using the Likert scale (Q16 and Q17). Scores for information sources are calculated based on the respondents’ answers using the Likert scale: 1 = Always; 2 = Sometimes; 3 = Rarely; 4 = Never; and 5 = Does not apply. The total response score was computed by adding up the points of the respondents’ selected answers for the 12 information sources, yielding a total score between 12 and 60, with high scores indicating more optimistic beliefs that individuals hold internally. The fourth section assesses the type of information gathered by the respondents. In the fifth and final section, the respondents were requested to fill a table to reflect their confidence and dependability level on sources of information using a three-level Likert scale and select the indicators they use for their trust in the information sources (Q17–Q20).

To overcome the language barriers of participants with different nationalities, the questionnaire was initially drafted in English, translated to Arabic to facilitate communication and understanding, and then back-translated to English to ease statistical analysis.

## DATA COLLECTION

A cross-sectional, closed-ended multiple-choice pilot survey was conducted on 442 residents of Qatar. An online self-reported questionnaire was used as a survey instrument. Invitations to participate in the study were distributed via different social media platforms.

Participants were selected voluntarily via online informed consent. The investigators first approached those who were available and easily accessible and sought their consent. Most of the study participants filled out the self-administered electronic questionnaire through a web link and requested to forward the questionnaire to their known eligible public members. A link to the questionnaire was also posted on the Hamad Medical Corporation website. This online approach was supplemented by in-person data collection.

The cross-sectional survey included individuals aged 18 years or older living in Qatar at the time of data collection. A total of 442 participants completed the questionnaire.

## DATA ANALYSES

The sample data was reviewed to remove outliers and missing data to ensure good quality. Analysis Toolpak statistical model in Excel was employed for data analyses. For all data collected descriptive statistics (mean, count, standard error, median, mode, standard deviation, sample variance, kurtosis, skewness, range, minimum, maximum, and sum) for continuous variables, whereas frequencies and percentages are used to describe the categorical variables. Logistic regression analyses were employed in this study for categorical variables. Data on determinants of COVID-19 infections were evaluated using percentages and a binary scale. Information sources were evaluated by Likert and ranked by Bloom’s scale.^12^

The association between 12 COVID-19 information sources and seven socioeconomic and demographic factors were determined using correlation analysis. This was done using a correlation matrix calculated to explore bivariate associations between the variables. Each of the seven factors (income level, education, sex, age, housing, occupation, marital status) is classified into subcategories (e.g., age divided into young, medium, and old). Following this, we calculated the significance of the effect of each category on the 12 different sources of information, and the quantity of preference score for each source of information. The accuracy of our calculations was confirmed through a chi-squared test.

## OUTCOME EVALUATION MEASURES

For evaluating the response to the questions using the Likert scale for each information source, the composite scores were calculated by relating the actual sum of scaled answers to the total sum of scores (five total number of respondents) for each source in each column. Likewise, composite scores were determined row-wise. These composite scores were ranked in descending order and evaluated using the Bloom’s cutoff score suggested by Alzahrani et al.^12^ based on the criteria: IF (the entry element of the composite score is >80, “it is evaluated as good,” and IF (the entry element >60, “ranked fair,” otherwise “it is ranked poor”)). For each data composite score, the interquartile range is determined. The association between sociodemographic characteristics (gender, age, marital status, educational level) and different sources of COVID-19 information was determined using correlation analysis. For implementing correlation analysis, the Likert scale data of each one of the seven socioeconomic variables with their respective subcategories was used as input for correlation analysis.

The assessment of the participants’ responses for determinant factors of infection by COVID-19, for example, receiving a vaccination, having an infection in the past, and the probability of being infected in the future, was evaluated for answering questions (Q8–Q15) of the questionnaire section two.

## RESULTS

Table 1 presents the descriptive statistics of respondents’ socioeconomic and demographic characteristics. Most of the participants were Single (264; 60%), The median age (IQR) was 32.4 years with a standard deviation of ±11 years, had a high education level (above secondary education), and most were non-Qatari residents. Approximately two-thirds of the respondents were females. The ages of community members were skewed toward the young and growing state (skewness 1.01), and they are at the economically active and growing stage (only 9% in the old category).

### Factors associated with a higher risk of COVID-19 infection occurrence

**Figure 1:**
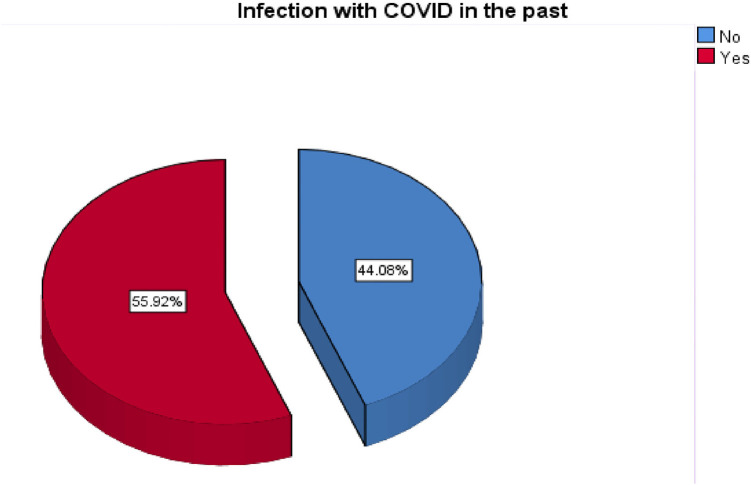
Depicts previous occurrences of COVID-19 infection.

Tables 2 and 3 shows that most of the housing is good and not crowded to aid in disease transmission. Other factors that might assist low transmission are (high vaccination—98%, Medium number of infections in the past—55%, as shown in Figure 1, and low level of susceptibility to a risk disease—11%).

These favorable conditions resulted in the participants’ perceptions of the effect and danger of infection with COVID-19 in the future being mild or low (71%), and the respondents viewed the disease as not fatal (33%).

The most reported chronic diseases associated with increased COVID-19 infection risk were diabetes mellitus (4%), followed by respiratory system disease (3%), and then hypertension (2%).

### The main sources of information

The frequency of consulted sources for information about coronavirus is given in Table 4. Using Bloom’s classification. The table revealed that traditional media, except for television (spouse/partner, newspaper (printed or internet), radio, and religious leaders), are least preferred, while fair preference is given to other sources of information.

The participants always preferred official governmental websites (39%), followed by family doctor/medical providers (25%), local mainstream media television (24%), official non-governmental websites (24%), and nonofficial social media platforms (22%).

### Type of COVID-19 information respondents want to know

Table 5 presents the kinds of information COVID-19 respondents want to know, i.e., looked-for information. Their order of knowledge preference in descending order is Travel restrictions (75%), COVID-19 new variants (60%), Symptoms of COVID-19 (59%), New development on vaccine and its potential safety (48%), information to help on how the respondent can personally prevent spread of the disease (47%).

### Trusted sources of information about COVID-19

Identification of the main and most trusted sources of information about COVID-19:

The identification of the main sources and most trusted sources of information about COVID-19 and sociodemographic and background factors are presented in Table 6. As presented in Table 6, COVID-19 information obtained from official governmental organizations and medical providers was the most trusted source, while non-governmental sources were least preferred. Information from personnel sources (spouse/partner, friends or coworkers, family members) is given more confidence than the traditional media (religious leaders, newspapers (printed or internet), radio, or podcasts) with the exception of television media, which is still a trusted source. However, confidence in the internet/social media/text messages is low (16%–18%).

### Factors associated with high trust in a given COVID-19 information source

The determining factors for personal trust and confidence in a source of information about COVID-19 are in Table 7. The majority of respondents reported that recommended information by healthcare professionals is highly trusted (89%), followed by sources that give facts supported by other sources and do not contradict them (47%), and then those sources that continuously updated (39%). Other indicators of confidence referred to in Table 7 are rated low.

### The association of sources of COVID-19 information and participants’ demographic profile

The association between 12 COVID-19 information sources, and seven socioeconomic and demographic factors were determined using correlation analysis. This was done using a correlation matrix calculated to explore bivariate associations between the variables. It can be observed that there is a weak association between age and partner (male/female; wife/husband) as a source of information.

The correlation coefficients between the multiple information sources are in the range of 0.508–0.694 and indicate weak strength and positive direction of the relationship between age, marital status, and income with the different information sources.

Association between age and frequency and use of family members, work colleagues, and local, land, and international television as a source of information is low to medium (0.659). There is a high association between age and family doctor, social, formal, non-governmental electronic sites, newspapers, and listening to broadcasts (0.659–0.753).

The three age classes are highly associated with all information sources (*r* = 0.755–0.946). Marital status association with all information sources is high. Likewise, the association between nationality is low, while it is high with Qatari and non-Qatari (*r* = 0.767–0.972). The association with income, occupation levels, and education is low, while its subcategories are high.

## DISCUSSIONS

The study suggests that the Ministry of Public Health (MOPH) is a key source of trusted information for the public in Qatar, with healthcare professionals playing an important role in promoting adherence to COVID-19 preventive measures. It provides basic evidence for creating evidence-based policies to control disease outbreaks, acknowledging that trust and information preferences may change over time.

The majority of participants used multiple sources for COVID-19, consistent with evidence that people rely on various information sources during pandemics.^13,14^ A study from Japan showed that the more information sources an individual uses, the more likely he or she is to adopt preventive measures. Prior research showed that individuals with low levels of understanding and concern about COVID-19 tend to use fewer information sources and are less likely to take protective action.

Using Bloom’s classification, the study revealed that traditional media, except for television (spouse/partner, newspapers (printed or internet), radio, and religious leaders), are least preferred, while fair preference is given to other sources of information. It seems that television plays an important role during pandemics; previous studies from the USA, Japan, and Iran also found television to be an important source of COVID-19 information.^15,17,18^

The participants always preferred official governmental websites and family doctor/medical providers; this is in agreement with results obtained in Iran and Japan.^15,17^ Among the communication channels examined in this study, MOPH was a widely consumed information source that might have the potential to shape public attitudes toward COVID-19 and enhance engagement in preventive actions.

The respondents ranked the kinds of information they wanted to know, giving the highest priority to information concerning Travel restrictions, followed by information related to new COVID-19 variants and disease symptoms. However, access to social services or resources is given less concern. This may be due to the high level of services made available by the Qatar health system.

Prior research has also identified that trust in information sources is an important predictor of preventive behavior.^19^ Thus, trust mediates the relationship between information use and health behaviors.^20–22^ In this study, COVID-19 information obtained from official governmental organizations and medical doctors was more trusted than from non-governmental sources. Information from personnel sources (spouse/partner, friends or coworkers, family members) is given more confidence than the traditional media (religious leaders, newspapers (printed or internet), radio, or podcasts) with the exception of television media, which is still a trusted and frequently used source. However, confidence in the internet/social media/text messages is low. These results are in agreement with studies from Saudi and Australia.

Regarding sources of COVID-19-related information, trusted sources and used sources always align. Healthcare professionals were highly trusted, and their usage was also high.

Key associations between trusted COVID-19 information sources and sociodemographic factors included marital status, occupation, and income. Most respondents trusted healthcare authorities more, suggesting that direct communication from healthcare professionals could be beneficial.

**Study Limitations:** The present study has several limitations. First, the representativeness of the study participants might have been affected by self-selection bias. Selection bias and sampling bias are common in online surveys.^25^ This is a pilot study with a cross-sectional design and a limited sample size. It is intended only to reflect initial results and evidence of the study objectives. This restricts the generalization of the findings to all Qatar residents. Hence, the study cannot confirm respondents’ preferences for COVID-19 information sources. Future research should examine changes in information-seeking behaviors over time and consider the probability of a larger sample size.

Finally, the participation in this survey is skewed toward non-Qatari-nationality as the participation of Qatari people is low. Recall that this is a pilot study, and it is then advised that more efforts be made to increase Qatari people’s participation in future work.

## CONCLUSIONS AND RECOMMENDATIONS

This pilot study revealed a significant disparity in COVID-19 information sources and preferences. The MOPH emerged as a widely used channel with the potential to influence the public’s adoption of preventive measures. Additionally, healthcare professionals were recognized as highly trusted sources, making them possibly essential for improving adherence to preventive measures.

In summary, encouraging access to multiple information sources, utilizing communication channels, and modifying messaging according to target group characteristics might be essential to promote COVID-19 preventive measures. While further research is required, these findings could contribute to encouraging the adoption of COVID-19 preventive measures.

## List of abbreviations

**Table:** 

MOPH	Ministry of Public Health
TV	Television
USA	United States of America

## Ethical clearance

All procedures performed in this study involving human participants complied with institutional and Qatar national research ethical standards. The study was approved by the Hamad Health Corporation Research Ethics Committee and was designed and performed according to the ethical principles established by the Corporation MRC-01-21-348.

## Data availability statement

The datasets used and/or analyzed during the current study are available from the corresponding author upon reasonable request.

## Conflicts of interest

All authors declare the following that no financial support was received from any organization for the submitted work. All authors have declared that they have no financial relationships with any organizations that might have an interest in the submitted work.

All authors have declared that there are no other relationships or activities that could appear to have influenced the submitted work.

## Authors’ contribution

The first author was in charge of designing, conducting the study, and writing the manuscript, while the second author assisted with and contributed to the conception and design of the study, supervised the study, and reviewed the manuscript. The third and fourth authors provided supervision for the project and interpretation of the data and contributed to the manuscript writing and participated in its revision. All authors gave approval to the final manuscript version to be submitted for publication.

## Acknowledgments

I acknowledge those whose contributions made this research study possible, and my supervisor, and my appreciation and thanks go to the physicians who helped collect the data for this research.

## Figures and Tables

**Table 1.** Frequency of respondents’ socioeconomic and demographic characteristics.

Socioeconomic	No	%
Sex	413	
Female	256	62
Male	157	38
Age	410	
<34	247	56
35–52	122	28
>52	41	9
Marital status	430	97
Married	264	60
Single	166	38
Nationality	437	
Qatari	9	2
Non-Qatari	428	97
Employment	380	
In health sector	137	31
In the non-health sector	140	32
Retired	6	1
Unemployed	97	22

**Table 2.** Shows past diagnosis with risk disease for COVID-19 infection occurrence.

**Type of disease**	**No**	**%**
Total respondents	441	100
With chronic health conditions	49	11.11
Without chronic health conditions	392	88.89
Hyperthyroid	2	0.45
Chron’s disease	1	0.23
Diabetes mellitus	18	4.08
Respiratory system disease	12	2.72
Hypertension	9	2.04
Psoriasis	1	0.23
Gout	1	0.23
Asthma	2	0.45
Coronary artery disease	1	0.23
Hypothyroidism	2	0.45

**Table 3.** Shows factors associated with COVID-19 infection occurrence and participants’ risk perception.

**Risk factors**	**No**	**%**
Took vaccination	431	98
Yes	411	93
No	20	5
Perceived probability of COVID-19 infection	439	99
High	104	24
Medium	20	5
Low	313	71
Evaluation of danger of infection	413	93
High	105	24
Medium	172	39
Low	147	33
Housing living condition	441	100
Single alone	147	33
With family and kids (age <18 years)	294	67
With crowdy group	0	0

**Table 4.** Frequency of consulted sources for information about coronavirus.

**Consulted source for coronavirus information**	**Always**		**Sometimes**		**Rare**		**Never**		**Not applicable**		**Composite score**	**Preference evaluation**
	No	%	No	%	No	%	No	%	No	%		
Spouse/partner	48	13	90	24	48	13	36	10	149	40	52	Poor
Other family members	57	15	143	39	100	27	45	12	26	7	69	Fair
Friends or coworkers	72	19	171	46	79	21	33	9	16	4	73	Fair
Local mainstream media television	88	24	134	36	80	22	40	11	29	8	71	Fair
International mainstream media	68	18	132	36	84	23	51	14	36	10	68	Fair
Doctor/medical provider	91	25	140	38	77	21	42	11	21	6	73	Fair
Official governmental website*s*	145	39	122	33	66	18	22	6	16	4	79	Fair
Official non-governmental websites	89	24	128	35	84	23	49	13	21	6	72	Fair
Nonofficial social media platforms	80	22	152	41	93	25	31	8	15	4	74	Fair
Newspaper (printed or internet)	37	10	97	26	88	24	94	25	55	15	58	Poor
Radio	43	12	76	20	91	25	100	27	61	16	57	Poor
Religious leaders	27	7	71	19	82	22	127	34	64	17	53	Poor

**Table 5.** Type of information respondents want to know.

**Responder choices**	**No**	**%**
Travel restrictions	274	75.27
New developments on vaccine and its potential safety	174	47.80
COVID-19 new variants	217	59.62
Treatments currently in use or in development	140	38.46
How I can personally prevent spread of the disease	170	46.70
Access to social services or resources	55	15.11
Caring for a person who is at risk	122	33.52
Information on my children’s education	69	18.96
Infection rates and mortality rates	142	39.01
Symptoms of COVID-19	216	59.34
Testing rates and procedures	118	32.42
Personal stories from others related to COVID-19	87	23.90
no search for COVID-19 information in last 6 months	80	21.98
Other (please specify)	9	2.47

The total number of responses was 364; no response was 78.

**Table 6.** Assess the trust and confidence in different COVID-19 information sources.

Sources of information	Great trust		Tend to trust it		Tend to distrust it		Distrust it greatly		Not sure or don’t know		Not applicable		Total	Weighted average
	No	%	No	%	No	%	No	%	No	%	No	%		
Spouse/partner	111	31	79	22	11	3	9	3	15	4	128	36	353	4.35
Other family members	103	29	160	45	33	9	17	5	17	5	23	7	353	3.30
Friends or coworkers	62	18	198	56	30	9	18	5	23	7	22	6	353	3.46
Official governmental	189	54	116	33	18	5	10	3	7	2	13	4	353	2.78
Official non-governmental websites	78	22	133	38	70	20	32	9	24	7	16	5	353	3.54
Nonofficial social media platforms	35	10	100	28	99	28	61	17	40	11	18	5	353	4.07
Doctor/medical provider	207	59	117	33	7	2	6	2	8	2	8	2	353	2.63
Local mainstream media television	101	29	157	44	33	9	10	3	30	9	22	6	353	3.37
International mainstream media television	62	18	157	44	45	13	31	9	34	10	24	7	353	3.69
Religious leaders	57	16	113	32	38	11	23	7	47	13	75	21	353	4.33
Newspapers (printed or internet)	64	18	140	40	34	10	17	5	33	9	65	18	353	4.03
Radio or podcasts	55	16	133	38	48	14	17	5	44	12	56	16	353	4.08

The total number of responses was 353; no response was 89.

**Table 7.** Factors employed to determine trust in information.

**Respondent choices**	**Responses**	
Factors employed to determine trust in information	No	%
Recommended by healthcare professionals	307	89
Recommended by a relative or friend	75	22
Recommended by media	82	24
Corresponds with facts from other sources	162	47
References are provided	134	39
Well-written language	72	21
Other people trust the given source	47	14
Continues updating	118	34

The total number of responses was 345; no response was 97.

## Appendix

**Questionnaire on Patterns of COVID-19 Information Sources and its Associated Factors Among the Public in Qatar: A Cross-Sectional Survey**

**(A) Socio-demographics:**

**Table:**

**1. Age (completed years)**		
**2. Gender:**	∘ Male	∘ Female
**3. Education level:**	∘ No formal education ∘ Primary ∘ Preparatory	∘ Secondary ∘ College/university ∘ Postgraduate studies ∘ Others
**4. Occupation:**	∘ Employed in health sector ∘ Employed in non-health sector	∘ Unemployed ∘ Retired
**5. Marital status:**	∘ Single ∘ Married	∘ Widow ∘ Divorce
**6. Nationality:**		
**7. Income:**	∘ Less than 5,000 QAR ∘ 5,000–15,000 QAR	∘ 15,000–30,000 QAR ∘ More than 30,000 QAR ∘ Not applicable
**8. Who lives in your household besides yourself? Choose as many as apply**	∘ I live alone ∘ I live with children under 18 ∘ I live with adults who are not in a COVID-19 risk group	∘ I live with people in a COVID-19 risk group (e.g., people over 65 years and/or with chronic disease) ∘ Others, please specify________________
**9. Do you have a chronic illness?**	∘ No	∘ Yes
**10. If yes to question no. 9, please specify**	∘ Diabetes mellitus ∘ Hypertension ∘ Immunocompromising conditions, e.g., cancer	∘ Respiratory system disease ∘ Others, please specify_______________
**11. Have you been infected with COVID-19?**	∘ No	∘ Yes
**12. Have you been vaccinated for COVID-19**	∘ No	∘ Yes

**(B) COVID-19 risk perception:**

**Table:**

**13. What do you consider to be your own probability of getting infected with COVID-19?**
Extremely unlikely [1] [2] [3] [4] [5] [6] [7] Extremely likely

**14. How susceptible do you consider yourself to an infection with COVID-19?**
Not susceptible [1] [2] [3] [4] [5] [6] [7] Very susceptible
**15. If you get infected with COVID-19, how seriously ill do you think you would be?**
Not severe [1] [2] [3] [4] [5] [6] [7] Very severe

**(C) COVID-19 information sources**

**Table:**

**16. How often have you searched for information about COVID-19 in the past 6 months?**
a. Multiple times a day
b. Once a day
c. Several times a week
d. Once a week
e. Less than once a week
f. I didn’t search for COVID-19-related information in the past 6 months.
g. If your answer to question 16 is F, kindly end the questionnaire, and we would like to thank you for your participation.

**Table:**

**17. Do you get information about Coronavirus from any of the below sources?**
17.1. Spouse/partner.
a. Always
b. Sometimes
c. Rare
d. Never
e. Not applicable
17.2. Other family members.
a. Always
b. Sometimes
c. Rare
d. Never
e. Not applicable
17.3. Friends or coworkers.
a. Always
b. Sometimes
c. Rare
d. Never
e. Not applicable
17.4. Local mainstream media television (e.g., Qatar TV, Al Jazeera, AlRayyan TV)
a. Always
b. Sometimes
c. Rare
d. Never
e. Not applicable
17.5. International mainstream media television (e.g., BBC, CNN)
a. Always
b. Sometimes
c. Rare
d. Never
e. Not applicable
17.6. Doctor/medical provider.
a. Always
b. Sometimes
c. Rare
d. Never
e. Not applicable
17.7. Official governmental (e.g., the MOPH, HMC, PHCC) websites or official social media platforms (e.g., MOPH Facebook page).
a. Always
b. Sometimes
c. Rare
d. Never
e. Not applicable
17.8. Official non-governmental websites or their social media platforms (e.g., WHO Facebook page).
a. Always
b. Sometimes
c. Rare
d. Never
e. Not applicable
17.9. Non-official social media platforms (e.g., Facebook, Twitter, WhatsApp) or websites (e.g., Wikipedia).
a. Always
b. Sometimes
c. Rare
d. Never
e. Not applicable
17.10. Newspapers (printed or internet, e.g., Alraya newspaper).
a. Always
b. Sometimes
c. Rare
d. Never
e. Not applicable
17.11. Radio.
a. Always
b. Sometimes
c. Rare
d. Never
e. Not applicable
17.12. Religious leaders.
a. Always
b. Sometimes
c. Rare
d. Never
e. Not applicable

**(D) Types of COVID-19 information searched by the public:**

**Table:**

What kinds of information related to the COVID-19 pandemic did you look for? Please select all applicable
□ Travel restrictions
□ Progress on development of a COVID-19 vaccine and potential vaccine safety
□ COVID-19 new variants
□ Treatments for COVID-19 currently in use or in development
□ How I can personally prevent the spread of the disease
□ Access to social services or resources
□ Caring for a person who is at risk
□ Information on my children’s education
□ Infection rates
□ Symptoms of COVID-19
□ Testing rates and procedures
□ Personal stories from others related to COVID-19
□ Other: **[PLEASE SPECIFY]** __________
□ I did not look for any information related to COVID-19 during the last 6 months

**(E) COVID-19 trustable information sources:**

For each of the following information sources, please indicate whether you tend to trust it or not trust it.

**Table:**

	Trust it a great deal	Tend to trust it	Tend to distrust it	Distrust it greatly	Not sure or don’t know	Not applicable
**a. Spouse/partner**	□	□	□	□	□	□
**b. Other family members**	□	□	□	□	□	□
**c. Friends or coworkers**	□	□	□	□	□	□
**d. Official governmental (e.g., the MOPH, HMC, PHCC) websites or official social media platforms (e.g., MOPH ‎Facebook page)**. **‎**	□	□	□	□	□	□
**e. Official non-governmental websites or their social media platforms (e.g., WHO Facebook page).‎**	□	□	□	□	□	□
**f. Non-official social media platforms (e.g., Facebook, Twitter, WhatsApp) or websites (e.g., Wikipedia).**	□	□	□	□	□	□
**g. Doctor/medical provider**	□	□	□	□	□	□
**h. Local mainstream media television (e.g., Qatar TV, Al Jazeera, AlRayyan TV)‎**	□	□	□	□	□	□
**i. International mainstream media television (e.g., BBC, CNN)‎**	□	□	□	□	□	□
**j. Religious leaders**	□	□	□	□	□	□
**k. Newspapers (printed or internet, e.g., the Alraya)**	□	□	□	□	□	□
**l. Radio or podcasts**	□	□	□	□	□	□

**(F) Factors for judging the trustability of different COVID-19 information sources:**

What are the important factors for you to trust a given COVID-19 information source? Please choose all applicable.

**Table:**

□ Recommended by healthcare professionals
□ Recommended by a relative or friend
□ Recommended by media
□ Corresponds with facts from other sources
□ References are provided
□ Well-written language
□ Other people trust the given source
□ Continues updating
□ Other: **[PLEASE SPECIFY]** __________
